# Verification of Pharmacogenetics-Based Warfarin Dosing Algorithms in Han-Chinese Patients Undertaking Mechanic Heart Valve Replacement

**DOI:** 10.1371/journal.pone.0094573

**Published:** 2014-04-11

**Authors:** Li Zhao, Chunxia Chen, Bei Li, Li Dong, Yingqiang Guo, Xijun Xiao, Eryong Zhang, Li Qin

**Affiliations:** 1 Department of Laboratory Medicine, West China Hospital, Sichuan University, Chengdu, P. R. China; 2 Department of Cardiothoracic Surgery, West China Hospital, Sichuan University, Chengdu, P.R. China; Technische Universität Dresden, Medical Faculty, Germany

## Abstract

**Objective:**

To study the performance of pharmacogenetics-based warfarin dosing algorithms in the initial and the stable warfarin treatment phases in a cohort of Han-Chinese patients undertaking mechanic heart valve replacement.

**Methods:**

We searched PubMed, Chinese National Knowledge Infrastructure and Wanfang databases for selecting pharmacogenetics-based warfarin dosing models. Patients with mechanic heart valve replacement were consecutively recruited between March 2012 and July 2012. The predicted warfarin dose of each patient was calculated and compared with the observed initial and stable warfarin doses. The percentage of patients whose predicted dose fell within 20% of their actual therapeutic dose (percentage within 20%), and the mean absolute error (MAE) were utilized to evaluate the predictive accuracy of all the selected algorithms.

**Results:**

A total of 8 algorithms including Du, Huang, Miao, Wei, Zhang, Lou, Gage, and International Warfarin Pharmacogenetics Consortium (IWPC) model, were tested in 181 patients. The MAE of the Gage, IWPC and 6 Han-Chinese pharmacogenetics-based warfarin dosing algorithms was less than 0.6 mg/day in accuracy and the percentage within 20% exceeded 45% in all of the selected models in both the initial and the stable treatment stages. When patients were stratified according to the warfarin dose range, all of the equations demonstrated better performance in the ideal-dose range (1.88–4.38 mg/day) than the low-dose range (<1.88 mg/day). Among the 8 algorithms compared, the algorithms of Wei, Huang, and Miao showed a lower MAE and higher percentage within 20% in both the initial and the stable warfarin dose prediction and in the low-dose and the ideal-dose ranges.

**Conclusions:**

All of the selected pharmacogenetics-based warfarin dosing regimens performed similarly in our cohort. However, the algorithms of Wei, Huang, and Miao showed a better potential for warfarin prediction in the initial and the stable treatment phases in Han-Chinese patients undertaking mechanic heart valve replacement.

## Introduction

Warfarin is one of the most widely prescribed anticoagulants for the prevention of thromboembolic events associated with atrial fibrillation, venous and arterial thrombosis, especially in patients of rheumatic heart disease with mechanic heart valve replacement. Because warfarin has a narrow therapeutic range and wide interindividual variability in treatment response, the traditional clinical treatment, i.e. experience-based dosing, may often lead to under or over dosages of warfarin. Low dose treatment may not reach effective anticoagulation and high dose may increase the risk of bleeding in the initial therapeutic phase [1]–[4]. Therefore, accurate medication in the initial stage of warfarin anticoagulation is very important for patients with mechanic heart valve replacement.

Since the discovery of the relationship between the gene polymorphisms of *VKORC1* and *CYP2C9* and the individual variability in warfarin dose requirements in the anticoagulation therapy [5]–[8], a number of pharmacogenetics-based algorithms integrating demographic data and different genotypes have been established to predict the dose of warfarin in Han-Chinese population [9]–[16] and other populations [17]–[21] all over the world. Despite numerous amounts of studies comparing the predictive accuracy of warfarin dosing regimens, nearly all of published papers mainly focused on assessing the performance of these algorithms in mixed populations and little data is available on studying the algorithms performance based on Han-Chinese population in patients undertaking heart valve replacement [22]–[24]. Because the Caucasian patients usually start warfarin at higher doses than the Han-Chinese [17], [25], the equations based on the Caucasian populations may not be suitable for predicting the dose of warfarin in the Han-Chinese patients. Besides, the pharmacogenetics-based warfarin dosing algorithms established by other diseases might not be appropriate for predicting the warfarin dose in patients with mechanic heart valve replacement.

In this study, we aimed to evaluate the performance of pharmacogenetics-based warfarin dosing algorithms built in Han-Chinese population and two authoritative algorithms, namely International Warfarin Pharmacogenetics Consortium (IWPC) algorithm [17] and the Gage *et al*. algorithm [18], in the initial and the stable warfarin treatment stages in a cohort of Han-Chinese patients undertaking mechanic heart valve replacement.

## Materials and Methods

### Ethics Statement

The study protocol was approved by the Ethics Review Board of West China Hospital of Sichuan University. The study conformed to the principles outlined in the declaration of Helsinki. All patients provided written informed consent before participating in the study.

### Study Design and Study Samples

All patients admitted for mechanic heart valve replacement were registered in our cohort in West China Hospital of Sichuan University from March 2012 to July 2012 and were followed up for 3 months after the surgery. Patients were excluded if they fulfilled the following criteria: 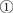 Age below 18 years; 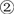 Ethnicity other than Han-Chinese; 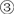 Patients taking aspirin during the warfarin therapy; 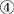 Patients with serious complications, including serious infections, renal impairment, heart failure, and respiratory failure; 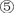 Patients with abnormal liver and coagulation function before the surgery.

Eligible patients were recruited 1 day prior to surgery and blood was collected for *VKORC1* (1173G>A, rs9934438) and CYP2C9*3 (1075G>T, rs1057910) genotyping. We collected the following information of these patients from the hospital information system and by telephoning the patients after discharge: 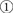 Demographic data: age, gender, weight, and height; 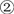 Other medications during initial warfarin therapy; 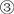 INR results during the follow-up period; 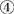 Warfarin doses (starting and maintenance warfarin dose); 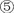 Smoking and drinking history.

The observed initial warfarin dose (starting warfarin dose) was defined as the dose that led to the value of International Normalized Ratio (INR) of the patient within the therapeutic range (INR = 1.5–2.5) for the first time. The actual stable dose of warfarin (maintenance warfarin dose) was the amount of warfarin required to achieve a stable INR (INR = 1.5–2.5) in 3 consecutive laboratory measurements separated by at least 1 week. Smoker was defined as self-reported use of tobacco products (use of cigarettes, pipes, chewing tobacco or snuff) every day and had smoked in the past 30 days at the time the study was conducted [26], [27]. In accordance with Chinese dietary standards, alcoholic was defined as a daily consumption of pure alcohol of 15 g or more in females and 25 g or more in males during the past 1 year [28].

### Genotyping

Two milliliters of venous blood sample were collected from the recruited patients 1 day prior to surgery. Genomic DNA was extracted by QIAamp DNA Blood mini kit (Qiagen, Germany), according to the manufacturer's instructions. The extracted genomic DNA was amplified by PCR with *VKORC1* specific primers (5′-CCGAGAAAGGTGATTTCCAA-3′ (Forward); 5′-TGACATGGAATCCTGAC GTG-3′ (Reversed)), and CYP2C9*3 specific primers (5′-CACGAGGTCCAGAGA TAC-3′ (Forward); 5′-GGAATGAGATAGTTTCTGAATTTAAT-3′ (Reversed)). The PCR reaction was performed with the use of BigDye Terminator v3.1 Cycle Sequencing Kit, according to the manufacturer's instructions (Applied Biosystems, USA). Amplified fragments were purified using the ABI Company's ethanol disposition method and sequenced on the ABI PRISMTM 3730xl DNA analyzer (Applied Biosystems, USA). Data were analyzed with the use of Sequencing Analysis software v5.2 (Applied Biosystems, USA). The genomic sequences of CYP2C9*3 and *VKORC1* obtained from GenBank (NC_000010.10, NC_000016.9 respectively) were used as references.

### Algorithms Selection and Evaluation

We searched the pharmacogenetics-based warfarin dosing algorithms based on Han-Chinese population and the Gage *et al.* algorithm, and IWPC algorithm by PubMed, Chinese National Knowledge Infrastructure (CNKI), Wanfang databases. Of importance, the selected warfarin dosing algorithms should include both basic clinical and genetic variables of at least two genes, CYP2C9*3 and *VKORC1*.

The predicted warfarin dose of each patient was calculated using the regression equations of the selected models by the collected clinical and genetic information. The IWPC equation of Klein *et al.* is provided in the supplementary appendix [17]. The predicted warfarin dose of the Gage *et al.* algorithm was calculated by the website of http://www.WarfarinDosing.org (last accessed on April 27, 2013), according to the website instructions. If an algorithm contained a *VKORC1* polymorphism which was not 1173G>A, but in strong linkage disequilibrium with it, genotyping results of *VKORC1* 1173G>A (rs9934438) were utilized, as described in the literature [6], [17].

The MAE and the percentage of patients whose predicted warfarin dose fell within 20% of the actual therapeutic dose were used to evaluate the predictive accuracy of pharmacogenetics-based warfarin dosing algorithms on the whole. MAE was defined as the average of differences between the predicted warfarin doses and the actual doses.

However, to further compare the performance of pharmacogenetics-based warfarin dosing algorithms, we divided warfarin dose range into 3 groups according to our experience and literature reports [6], [23], [29], [30]: a low-dose group (<1.88 mg/day), an ideal-dose group (1.88–4.38 mg/day), and a high-dose group (>4.38 mg/day). Percentage of patients whose predicted warfarin dose was below 20% of the actual dose (underestimation), within 20% of the actual dose (ideally dosed), and above 20% of the actual dose (overestimation) were calculated to assess the predictive accuracy of every algorithm in the 3 warfarin dose groups.

### Statistical Analysis

Descriptive statistics were performed to determine frequency distribution, percentage distribution, mean and standard deviations. Correlations between the therapeutic warfarin dose (actual initial and stable warfarin dose) and genotypes were assessed using one-way ANOVA, and independent samples *t* test was used when the comparison was conducted between two groups. All the statistical analysis was performed by SPSS 16.0. The calculation of MAE and predicted percentages was performed in excel spreadsheet. *P*<0.05 was considered significant and all the statistical tests were two sides.

## Results

### Characteristics of the Study Population

A total of 194 patients received mechanic heart valve replacement in our hospital during study period, from March 2012 to July 2012. Out of the 194 patients, four patients were not Han nationality, one patient was less than 18 years old, five patients suffered from serious infection and heart failure after the surgery, and three patients died during the 3 months follow-up. After excluding these ineligible patients, a total of 181 patients were included in the analysis. Among the 181 patients, one hundred twenty-two patients reached the stable warfarin therapeutic range during the 3 month follow-up visit. The clinical, genetic, combination medication, smoking, and drinking characteristics of the patients were summarized in Table 1.

**Table 1:** **Characteristics of study population.**

Variables	Patients(n = 122)
Female, n (%)	84 (68.85)
Age, years, mean (SD)	50.25 (9.78)
Weight, kg, mean (SD)	58.9 (9.42)
Height, cm, mean (SD)	159.5 (10.54)
Smoking, n (%)	24 (19.67)
Drinking, n (%)	12 (9.84)
Amiodarone, n (%)	48 (39.34)
Digoxin, n (%)	47 (38.5)

### Associations between Genetic Variables and Warfarin Dose

**Figure 1:**
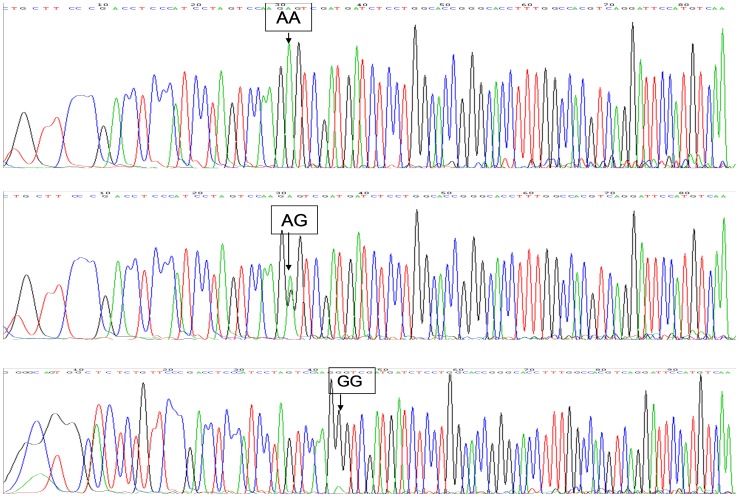
Genotyping results of the gene of *VKORC1.* AA represents the AA genotype of *VKORC1* gene. AG represents the AG genotype of *VKORC1* gene. GG represents the GG genotype of *VKORC1* gene.

**Figure 2:**
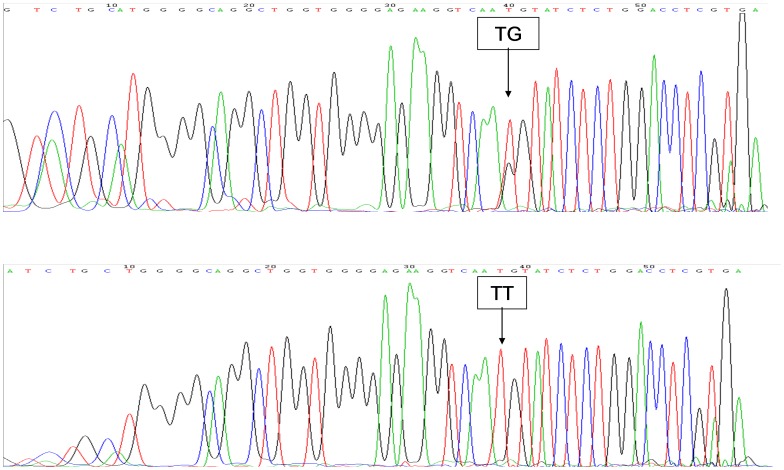
Genotyping results of the gene of CYP2C9*3. TG represents the TG genotype of CYP2C9*3 gene. TT represents the TG genotype of CYP2C9*3 gene.

The genotyping results of the two genes of *VKORC1* and CYP2C9*3 were given in Figure 1 and Figure 2, respectively. A summary of the relationships between genotypes and warfarin starting and maintenance dose was shown in Table 2. Warfarin dose differed significantly across genotypes for CYP2C9*3 (*P* = 0.001) and *VKORC1* (*P* = 0.018) in the actual initial treatment stage and in the stable warfarin treatment stage (CYP2C9*3 (*P* = 0.000) and *VKORC1* (*P = *0.011), respectively).

**Table 2:** The associations between genotypes and warfarin dose (mean±SD, mg/day).

Genotyping	n	Actual stable warfarin dose	Actual initial warfarin dose	P value
*VKORC1*	122	2.48±0.85	2.49±0.91	
AA	103 (84.4%)	2.32±0.59	2.33±0.60	0.011*
AG	18 (14.8%)	3.18 ±1.25	3.14±1.29	0.018**
GG	1 (0.80%)	6.25	7.5	
*CYP2C9* * *3*	122	2.48±0.85	2.49±0.91	
TT	103 (84.4%)	2.60±0.85	2.62±0.92	0.000*
TG	19 (15.6%)	1.83±0.52	1.85±0.52	0.001**

* represented the comparison of actual stable wafarin dose in different genotypes for the gene polymorphisms of VKORC1 and CYP2C9*3.

** represented the actual initial warfarin dose comparison in different genotypes for the gene polymorphisms of VKORC1 and CYP2C9*3.

### Selected Warfarin Dosing Algorithms

Six algorithms based on Han-Chinese population including Miao *et al.*, Huang *et al.*, Wei *et al.*, Zhang *et al.*, Lou *et al.*, and Du *et al.* met our requirements [9]–[14]. IWPC [17] and the Gage *et al.* algorithms [18], which were considered the authoritative algorithms in warfarin pharmacogenomics, were also included in our study. Specific algorithms were listed in Table 3.

**Table 3:** Algorithms selected for analysis.

Algorithm	Population	Target INR	Clinical variables	R^2^	Ref
Du *et al.*	Han	1.5–3.0	age, weight, *CYP2C9*, *VKORC1*	0.550	[14]
Huang *et al.*	Han	1.8–3.0	age, BSA, *CYP2C9*, *VKORC1*	0.541	[10]
Miao *et al.*	Han	1.5–3.0	age, weight, *CYP2C9*, *VKORC1*	0.628	[9]
Wei *et al.*	Han	1.5–3.0	age, weight, PTE, β-blocker,	0.517	[11]
			AMIO, CYP2C9*3, *VKORC1*, *CYP4F2*		
Zhang *et al.*	Han	2.0–3.0	age, weight, *CYP2C9*, V*KORC1*	0.671	[12]
Lou *et al.*	Han	1.5–3.0	age, weight, height, digoxin, amiodarone,	0.652	[13]
			*CYP2C9*, *VKORC1*, *CYP4F2*		
Gage *et al.*	mixed	2–2.8 and1.5–2.0	*CYP2C9*, *VKORC1*, BSA, target INR,	0.531	[18]
			smoking, race, PTE, amiodarone, age		
IWPC	mixed	2.0–3.0	age, height, weight, race, liver function,	0.314	[17]
			enzyme inducer, amiodarone,		
			*CYP2C9*, *VKORC1*		

### Evaluation of Algorithms Performance

Overall, the pharmacogenetics-based dosing algorithms could predict warfarin dose requirements in the entire cohort with the average percentage within 20% of 54%±7% (45%–66%) and 56%±8% (45%–67%) in the initial and the stable warfarin treatment phases, respectively. The MAE of all of the selected algorithms was less than 0.6 mg/day in both the initial and the stable warfarin dose predictions with the respective average values of 0.28±0.14 mg/day (0.003–0.58 mg/day) and 0.29±0.14 mg/day (0.02–0.59 mg/day). However, among the identified algorithms, the models of Wei *et al*., Huang *et al.*, and Miao *et al.* tended to perform better than others, with respective percentages within 20% of 66%, 58%, and 61% and MAE of 0.003 mg/day, 0.06 mg/day, and 0.18 mg/day in the initial warfarin treatment phase, and percentages within 20% of 67%, 61%, and 64%, and MAE of 0.02 mg/day, 0.07 mg/day, 0.19 mg/day in the stable warfarin treatment phase. By contrast, the Gage *et al.* and IWPC algorithms predicted the warfarin dose with average MAE of 0.37(0.24–0.50) mg/day and 0.52(0.39–0.64) mg/day and percentage within 20% of 52% and 47% in the initial treatment stage, and 0.38(0.26–0.51) mg/day and 0.53(0.41–0.65) mg/day and 53% and 49%, respectively, in the stable treatment stage. All the results were summarized in Table 4 and Table 5.

**Table 4:** Prediction evaluation of warfarin dose in initial stage (n = 122).

Algorithm	Underestimation (%)	Ideal (%)	Overestimation (%)	MAE(95%CI) mg/day	Ref
Du *et al.*	0.09	0.52	0.39	0.26(0.12–0.40)	[14]
Huang *et al.*	0.16	0.58	0.26	0.06(-0.07-0.18)	[10]
Miao *et al.*	0.09	0.61	0.30	0.18(0.01–0.35)	[9]
Wei *et al.*	0.10	0.66	0.24	0.003(-0.13-0.13)	[11]
Zhang *et al.*	0.09	0.52	0.39	0.24(0.09–0.39)	[12]
Lou *et al.*	0.07	0.45	0.48	0.58(0.43–0.72)	[13]
Gage *et al.*	0.05	0.52	0.43	0.37(0.24–0.50)	[18]
IWPC	0.04	0.47	0.49	0.52(0.39–0.64)	[17]
Mean±SD	0.09±0.04	0.54±0.07	0.37±0.10	0.28±0.14	

**Table 5:** Prediction evaluation of warfarin dose in stable stage (n = 122).

Algorithm	Underestimation (%)	Ideal (%)	Overestimation (%)	MAE(95%CI) mg/day	P value	Ref
Du *et al.*	0.08	0.53	0.39	0.27(0.14–04)	0.885	[14]
Huang *et al.*	0.14	0.61	0.25	0.07(-0.11-0.13)	0.878	[10]
Miao *et al.*	0.08	0.64	0.28	0.19(0.02–0.36)	0.912	[9]
Wei *et al.*	0.09	0.67	0.24	0.02(-0.1-0.14)	0.882	[11]
Zhang *et al.*	0.08	0.52	0.40	0.25(0.11–0.39)	0.896	[12]
Lou *et al.*	0.05	0.45	0.50	0.59(0.45–0.74)	0.898	[13]
Gage *et al.*	0.04	0.53	0.43	0.38(0.26–0.51)	0.884	[18]
IWPC	0.03	0.49	0.48	0.53(0.41–0.65)	0.877	[17]
Mean±SD	0.07%±0.04	0.56±0.08	0.37±0.10	0.29±0.14		

P value represented the comparison between initial and stable warfarin dose in the MAE. P<0.05 was statistically different.

When patients were stratified according to the initial and stable warfarin dose range, few patients were in a high-dose group. The majority of algorithms were significantly less predictive in the low-dose range (<1.88 mg/day) than in the intermediate-dose range (1.88–4.38 mg/day). Wei et al., Huang et al. and Miao et al. algorithms performed even better with percentages within 20% in the ideal-dose range prediction of 80%, 70%, and 81%, respectively, in the initial treatment phase and 80%, 70%, and 84%, respectively, in the stable treatment phase. However, the performances of Gage et al. and IWPC algorithms were relatively poor, with the respective percentages within 20% in the ideal-dose range prediction of 63% and 59% in the initial warafrin dose prediction and 69% and 57%, respectively, in the stable warfarin dose prediction. Specific results were shown in Table 6 and Table 7.

**Table 6:** Comparison of algorithms in the initial warfarin dose range (n = 122, %).

	Dose<1.88 mg/day (n = 35)			1.88<dose<4.38 mg/day (n = 82)			Dose>4.38 mg/day (n = 5)		
Algorithm	Under	Ideal	Over	Under	Ideal	Over	Under	Ideal	Over
Du *et al.*	3	17	80	7	70	23	40	60	0
Huang *et al.*	9	34	57	15	70	15	80	20	0
Miao *et al.*	0	17	83	10	81	9	80	20	0
Wei *et al.*	3	40	57	9	80	11	80	20	0
Zhang *et al.*	0	26	74	10	65	25	60	40	0
Lou *et al.*	14	26	60	2	52	46	20	80	0
Gage *et al.*	0	20	80	4	63	33	40	60	0
IWPC	6	26	68	1	59	40	40	60	0

**Table 7:** Comparison of algorithms based on the stable warfarin dose range (n = 122, %).

	Dose<1.88 mg/day (n = 35)			1.88<dose<4.38 mg/day (n = 82)			Dose>4.38 mg/day (n = 5)		
Algorithm	Under	Ideal	Over	Under	Ideal	Over	Under	Ideal	Over
Du *et al.*	0	21	79	6	69	25	80	20	0
Huang *et al.*	6	41	53	14	70	16	60	40	0
Miao *et al.*	0	21	79	7	84	9	80	20	0
Wei *et al.*	0	44	56	8	80	12	80	20	0
Zhang *et al.*	0	26	74	8	64	28	60	40	0
Lou *et al.*	12	26	62	3	49	48	0	100	0
Gage *et al.*	0	15	85	2	69	29	40	60	0
IWPC	3	29	68	1	57	42	40	60	0

## Discussion

In the present study, we compared the performance of pharmacogenetics-based warfarin dosing algorithms built in Han-Chinese population in a cohort of patients with mechanic heart disease. Because the Gage *et al.* [18] and IWPC algorithms [17] were derived from large sample sizes and mixed populations, and could adjust for race and various factors, they were also included in our study. Our study showed that nearly all of the selected pharmacogenetics-based warfarin dosing algorithms performed similarly in predicting the warfarin dose requirements in the initial and the stable treatment phases in the entire cohort. However, among the 8 algorithms compared, the algorithms of Huang *et al.*, *Miao et al.*, and Wei *et al.* performed better in both the initial and the stable warfarin dose predictions and in the low-dose and the ideal-dose ranges. Compared with the 3 models in Han-Chinese population, the Gage *et al.* and IWPC algorithms performed relatively poorly.

The Wei *et al.* algorithm could predict the highest proportion of predicted warfarin dose within 20% of the actual dose in the initial and the stable treatment phases on the whole. In the subgroup analysis, most algorithms tended to perform better in the ideal-dose group (1.88–4.38 mg/d) than in the low-dose group (<1.88 mg/d). Similarly, the 3 models of Huang *et al.*, *Miao et al.*, and Wei *et al.* performed well in the ideal- and low-dose prediction. Huang *et al.* algorithm was able to predict 70% of patients within 20% of their therapeutic dose in the ideal-dose range prediction in both the initial and the stable treatment phases, a proportion that is bigger than 47.5% (44.6–50.4%) obtained by Shin and Cao [23] and ∼50% reported by Liu [31]. Miao *et al.* algorithm, which was considered to perform poorly in predicting the warfarin dose in other papers [23], [29], [32], predicted the largest proportion of patients with the percentages within 20% in the ideal-dose range prediction of 81% and 84% in the initial and the stable dose treatment phases, respectively. Likewise, Wei *et al.* algorithm could predict 80% patients within 20% of their therapeutic dose in the ideal-dose range prediction in both the initial and the stable treatment phases. It also produced a higher predictive proportion in the low-dose range prediction with percentages within 20% of 40% in the initial treatment phase, and 44% in the stable treatment stage, which were the highest proportions in the low-dose range evaluation.

Additionally, despite the similarity with the percentage within 20% of the therapeutic dose reported in Asian people [29], [31], the Gage *et al.* and IWPC algorithms provided relatively poorer prediction than the 3 models of Wei *et al.*, Huang *et al.*, and Miao *et al.* in our study, with the value of 52% and 53% for the Gage *et al.* algorithm and 47% and 49% for IWPC algorithm in the initial and the stable warfarin dose predictions, respectively. Similarly, the predictive accuracy of the two algorithms in the subgroup analysis was also poor. The difference in the predictive accuracy of the 8 algorithms could be caused by: 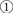 Difference in variables included in the equations; 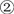 Difference in diseases of patients included in the model construction; 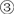 Different ethnicity of the study populations.

Genotyping of *VKORC1* and *CYP2C9* is recommended for adjusting warfarin doses [33]. Actually, small randomized controlled trials and systematic reviews have yielded promising results that patients receiving genotype-guided warfarin dosage required minor changes in dose adjustments [30], [34]. A recent case-control study conducted by Pirmohamed M *et al*. demonstrated that the pharmacogenetics-based dosing enabled patients starting warfarin for anticoagulation to reach the therapeutic window earlier than the standard dosing regime and was associated with the fewer incidences of excessive anticoagulation [35]. A study by Stephen E. Kimmel *et al.* also indicated the average time to reach the therapeutic range was shorter in the genotype-guided group than in the clinically guided group among black patients in the first 4 weeks warfarin therapy [36]. Importantly, our study likewise shows that the pharmacogenetics-based dosing algorithms could predict the warfarin dose with a high predictive accuracy. Therefore, the application of genotype-based warfarin dosing may be appropriate to help adjust warfarin doses to improve the effectiveness and reduce bleeding risks in warfarin anticoagulation therapy. Pharmacogenetics-based warfarin dosing is not practiced in China. Thus, the initial warfarin dose is usually decided on the basis of experience. Because of the potential cost of genotyping, clinical trials must be carried out before widespread adoption of pharmacogenetics-based warfarin dosing. In our study, we also found some patients' warfarin dose difference between the initial and the stable warfarin dose stages was as large as 1 mg/day. So, closely monitoring of INR is still very important even if a warfarin pharmacogenetics-based algorithm is used.

There are some limitations in our study. First of all, there were only 122 patients falling into the stable therapeutic range among the 181 patients followed up, leading to a relatively insufficient sample size. Secondly, we only compared the performance of the models based on Han-Chinese population in patients with mechanic heart valve replacement, further studies should be carried out to validate the performance of the algorithms in other ethnicities and other diseases. Thirdly, few patients were in the high-dose group in the subgroup analysis, we just compared the predictive accuracy of the selected models in the low- and ideal-dose group, resulting in the missing results in predicting the high dose of warfarin of the selected algorithms. Therefore, in the next work, larger sample size studies are needed to further confirm our results. Finally, our study is an observational study. Although our study showed that pharmacogenetics-based warfarin dosing algorithm could predict warfarin doses with desirable accuracy, controlled trails should be conducted to evaluate the effectiveness of pharmacogenetics-based warfarin dosing in the prevention of thromboembolic events, as well as major bleedings.
